# Daily intake of fermented milk with *Lactobacillus casei* strain Shirota reduces the incidence and duration of upper respiratory tract infections in healthy middle-aged office workers

**DOI:** 10.1007/s00394-015-1056-1

**Published:** 2015-09-29

**Authors:** Kan Shida, Tadashi Sato, Ryoko Iizuka, Ryotaro Hoshi, Osamu Watanabe, Tomoki Igarashi, Kouji Miyazaki, Masanobu Nanno, Fumiyasu Ishikawa

**Affiliations:** 1 Yakult Central Institute, 5-11 Izumi, Kunitachi-shi, Tokyo 186-8650 Japan; 2 Faculty of Research and Development, Yakult Honsha, Shinbashi, Tokyo 105-8660 Japan

**Keywords:** Probiotics, *Lactobacillus casei* strain Shirota, Upper respiratory tract infection, Common cold, NK cell activity, Cortisol

## Abstract

**Purpose:**

Although several studies have demonstrated the efficacy of probiotics for preventing upper respiratory tract infections (URTIs) in at-risk populations, including children and the elderly, few studies have investigated the efficacy of probiotics in healthy adults living normal, everyday lives. Thus, we tried to evaluate the effects of *Lactobacillus casei* strain Shirota-fermented milk (LcS-FM) on the incidence of URTIs in healthy middle-aged office workers.

**Methods:**

In a randomized controlled trial, 96 eligible male workers aged 30–49 years consumed LcS-FM containing 1.0 × 10^11^ viable LcS cells or control milk (CM) once daily for 12 weeks during the winter season. URTI episodes were evaluated by a physician via a questionnaire of URTI symptoms.

**Results:**

The incidence of URTIs during the intervention period was significantly lower in the LcS-FM group than in the CM group (22.4 vs. 53.2 %, *P* = 0.002). The time-to-event analysis showed that the LcS-FM group had a significantly higher URTI-free rate than the CM group over the test period (log-rank test: *χ* ^2^ 11.25, *P* = 0.0008). The cumulative number of URTI episodes and cumulative days with URTI symptoms per person was lower in the LcS-FM group, and the duration per episode was shorter. Inhibition of both reductions in NK cell activity in peripheral blood mononuclear cells and increases in salivary cortisol levels was observed in the LcS-FM group.

**Conclusion:**

The results suggest that the daily intake of fermented milk with LcS may reduce the risk of URTIs in healthy middle-aged office workers, probably through modulation of the immune system.

## Introduction

The human intestinal tract harbors more than 100 trillion bacteria, and the commensal gut microbiota plays a pivotal role in maintaining the health of the host [1]. Thus, much attention has been given to probiotics, which can survive the intestinal tract and recover/maintain balanced gut microbiota when ingested orally [2, 3]. Some strains of lactobacilli and bifidobacteria are popular probiotics, usually consumed as fermented dairy products or supplements [4]. Among the health benefits of probiotics, the prevention or control of infectious diseases is one of the most promising targets [5, 6]. Several studies have demonstrated that some probiotics are effective against not only infections in the gastrointestinal tract, but also those in the respiratory tract [7, 8].

Many clinical trials against upper respiratory tract infections (URTIs), such as the common cold and influenza, have evaluated various probiotic strains, and many of these have demonstrated efficacy against URTIs [7, 8]. The target populations in these clinical trials have generally been infants, children, students, and the elderly [9–12]. Since the immune defenses in these populations are relatively weak, the use of probiotics might be pertinent. However, few studies have investigated the efficacy of probiotics in healthy adults [13, 14].

Maintaining the immune defense system within a normal healthy state lowers the risk of URTIs. NK cell activity and salivary immunoglobulin A (IgA) are considered important in the prevention of URTIs [15, 16]. However, several environmental factors, including a stressful lifestyle, are likely to weaken the immune defense system [17], which may result in an increase in the risk of URTIs. There is some evidence to suggest that some probiotic strains restore NK cell activity and salivary IgA levels [18–20]. Thus, daily ingestion of a probiotic beverage might maintain normal immune function and control URTIs.

*Lactobacillus casei* strain Shirota (LcS) is a probiotic that can survive within the intestinal tract and recover balanced gut microbiota [21]. The immunomodulatory activities of the strain have been studied extensively in animal models and human studies [18, 22]. Studies in healthy subjects with low NK cell activity showed that ingestion of LcS-fermented milk (LcS-FM) recovered the activity [18]. Clinical trials have shown that LcS effectively reduces the risk of bladder cancer and colorectal tumor recurrences, and it has been proposed that immunomodulatory activities, including the recovery of NK cell activity, are among the underlying mechanisms [23]. Therefore, accumulating evidence implies that this probiotic has potential use as an immunostimulatory food material.

Some clinical trials with LcS have targeted URTIs. A double-blind, randomized, placebo-controlled trial in healthy elderly people attending day care facilities showed that consumption of LcS-FM reduced the duration of each URTI episode, but not the incidence rate [24]. Another trial conducted in healthy elderly nursing home residents showed a nonsignificant decreasing trend in the incidence of upper respiratory symptoms via LcS-FM ingestion [25]. On the other hand, a study conducted among healthy young athletes of endurance-based sports showed that LcS was effective in preventing URTIs [20]. In the latter study, daily consumption of LcS-FM significantly reduced the incidence of URTIs and the cumulative number of URTI episodes. Variations in the efficacy of LcS-FM might be due to differences among study populations.

We think that it is important to evaluate the efficacy of LcS in normal healthy adults, because events in daily life can lead to weakened immune defense responses. Thus, healthy middle-aged office workers were selected for this study. We measured the effect of the daily consumption of LcS-FM on the incidence of URTIs as the primary outcome. Additionally, we analyzed both NK cell activity and salivary IgA levels as immunological markers, and the levels of cortisol in saliva as a stress marker.

## Materials and methods

### Participants

Healthy male workers living in Tokyo or its suburbs, aged 30–49 years, and working within office buildings were recruited for this study via Web site advertising. The exclusion criteria were as follows: (1) working outside the office building twice or more a week; (2) difficulty providing saliva and blood samples; (3) pollinosis, chronic rhinitis, asthma, or milk allergy; (4) periodontitis or gingivitis; (5) history of serious liver, kidney, heart, lung, or gut disease; (6) receiving current medical treatment; (7) regularly consuming probiotics or fermented milk; (8) taking drugs or supplements that might affect the outcome of the study; (9) history of influenza vaccination or infection within the last 6 months; and (10) being deemed ineligible for this study by a physician, based on blood chemistry, blood pressure, pulse rate, or other reasons.

Participants were given a detailed explanation of the purpose and potential risks of the study, and 217 workers provided written consent and participated in a screening test, in which blood, urine, and saliva analysis, a physical examination, measurements of blood pressure and pulse, and a questionnaire regarding lifestyle and working profile were performed. Seventy-eight participants met the exclusion criteria, 10 with salivary IgA concentrations of 509 μg/ml or more were excluded, and 29 declined to participate prior to allocation. The remaining 100 participants were randomly allocated to two groups: the LcS-FM group and the control milk (CM) group.

### Sample size calculation

The sample size was calculated by assuming that the incidence of URTIs during the test period of 12 weeks would be 80 % and LcS-FM would reduce the incidence by 35 % [13, 20, 26]. At a significance level of 0.05 with 80 % power, it was estimated that 44 participants were needed per group to detect significant differences between the groups by the Chi-square test. Thus, 100 participants were included in this study.

### Study design

We conducted a randomized, controlled trial from December 8, 2012, through March 5, 2013, at the Chiyoda Paramedical Care Clinic (Tokyo, Japan). The study was conducted according to the guidelines laid down in the Declaration of Helsinki. All procedures were approved by the Institutional Review Board of the Chiyoda Paramedical Care Clinic. Written, informed consent was obtained from all participants.

### Test drinks

LcS-FM consisted of skimmed milk, high-fructose corn syrup, sugar, flavoring, and a minimum of 1.0 × 10^11^ live LcS cells. Probiotic strain LcS (YIT 9029) was obtained from the Culture Collection Research Laboratory of the Yakult Central Institute. Milk was used as a control beverage [12]. LcS-FM and CM drinks were placed into plastic bottles. The test drinks were delivered to each participant once a week under refrigeration and stored in a refrigerator until consumption.

### Procedures

Participants were allocated randomly to either the LcS-FM or CM group. Both groups were asked to consume one bottle of the test drink every day for 12 weeks and to refrain from consuming any other probiotic foods and supplements.

Participants kept a health diary during the intervention period each day before bedtime, recording body temperature, any symptoms of illness, and the impact of any symptoms on their daily activities by rating the impact as none/light, moderate, or severe. The test drinks consumed and all prohibited foods and supplements were also recorded in the diary. Participants were also asked to record possible symptoms of URTIs on the URTI symptom questionnaire on a daily basis.

Participants visited the clinic at weeks 0, 6, and 12 during the intervention period, and saliva and blood samples were collected. Participants were free to consult any doctor of their choice when experiencing symptoms of an illness, but were required to record the details of any diagnosis provided, hospital care given, prescribed and non-prescribed medications taken, and the results of influenza virus tests via influenza diagnosis kits if applicable in the health diary.

### Evaluation of URTIs

URTI episodes were evaluated by a physician at the clinic based on the information recorded in the health diary, the URTI symptom questionnaire, and interviews at 6 and 12 weeks after the intervention period commenced [12, 20]. The URTI symptom questionnaire consisted of questions on the following 16 symptoms: (1) fever; (2) chill; (3) headache; (4) runny nose; (5) stuffy nose; (6) sneezing; (7) cough; (8) sore throat; (9) sputum; (10) malaise; (11) muscular pain; (12) joint pain; (13) nausea; (14) diarrhea; (15) stomach ache; and (16) itchy eyes. The non-numerical ratings of none/light, moderate, and severe were scored as 1, 2, and 3, respectively, for each item. The daily symptom severity score for URTIs was calculated by summing all the scores for each item, except those for nausea, diarrhea, stomach ache, and itchy eyes, which were set to distinguish a URTI episode from gastrointestinal infections and pollinosis. Thus, the maximum score for daily symptom severity was 36. Symptoms separated by more than 2 consecutive symptom-free days were recorded as separate episodes.

Influenza was diagnosed according to the results of influenza virus test kits conducted as required at any hospital and was counted as a URTI episode. URTIs other than influenza were recorded as the common cold.

### NK cell activity

Peripheral blood mononuclear cells were freshly isolated, and NK cell activity was measured by the chromium release assay using K562 target cells. Peripheral blood mononuclear cells and ^51^Cr-labeled K562 cells were incubated at a ratio of 20:1 for 3.5 h, and radioactivity released from lysed target cells was measured. The percentage of specific lysis was calculated as NK cell activity using the following formula: specific lysis (%) = (experimental release − spontaneous release)/(maximal release − spontaneous release) × 100.

### Saliva analysis

Saliva was collected between 09:00 and 11:00 at the clinic by the passive drool method. Participants were asked to allow saliva to pool in the mouth for 3 min before transferring it via a straw to a collection tube. Saliva collection was repeated four times with 1-min intervals. The pooled saliva was centrifuged at 1500×*g* for 15 min, and the supernatant was stored below −20 °C until analysis.

The levels of secretory IgA and cortisol were determined by immunoassay kits (Salimetrics, PA) according to the instruction manuals.

### Adverse events

General biochemical and hematological testing of blood, urinary qualitative examinations, and physiological tests were performed at 0 and 12 weeks, and the results before and after the intervention period were compared. Any adverse health events were recorded by participants in their health diaries during the intervention period and confirmed by the clinic physician at weeks 6 and 12.

### Statistical analyses

The difference in URTI incidence between the groups was analyzed by the Chi-square test. Time-to-episode curves for the first URTI in the groups were described by the Kaplan–Meier method, and the difference was analyzed by the log-rank test (27). A hazard ratio was estimated using unadjusted Cox proportional hazard model. The risk reduction efficacy was calculated using the following formula: efficacy (%) = (1 − hazard ratio) × 100. Comparisons of the cumulative numbers of URTI episodes per person and levels of symptom severity between the groups were performed by the Wilcoxon two-sample test. Comparisons of cumulative days with symptoms per person and the durations of URTI episodes were achieved using the unpaired Student’s *t* test. Unpaired and paired Student’s *t* tests were, respectively, used for intergroup and intragroup comparisons of NK cell activity, salivary IgA secretion, and cortisol levels. Two-tailed *P* values <0.05 were considered statistically significant. All analyses were performed using IBM SPSS software version 20.0 (IBM Japan, Tokyo, Japan) and SAS software version 8.2 (SAS Institute Japan, Tokyo, Japan).

## Results

### Participant flow and characteristics

**Fig. 1:**
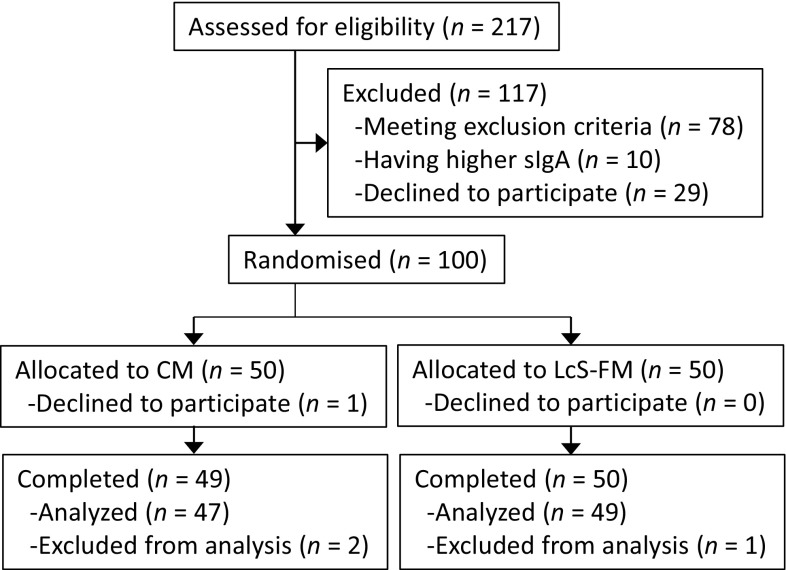
Participant flow diagram for this study

The participant flow diagram for this study is shown in Fig. 1. Eligibility was assessed in 217 male workers. Among them, 100 participants were recruited to the study and allocated to either the LcS-FM or CM group. One participant in the CM group did not start the intervention due to personal reasons, and 99 participants completed it. Of the 99 participants, 3 were excluded from the analysis for the following reasons: one started medication for hypertension during the intervention period; one consumed prohibited probiotic drinks on 10 days during the test period; and one was found to meet an exclusion criterion of the study. Thus, 96 participants comprising 49 participants in the LcS-FM group and 47 participants in the CM group were used for data analysis.

The baseline characteristics of the 96 eligible participants from the two groups are summarized in Table 1. No significant differences in age, body mass index, living with a child, smoking habits, or salivary IgA levels were found between the groups. Both groups showed very good compliance in terms of test drink consumption.

**Table 1:** Baseline characteristics of participants

	CM (*n* = 47)		LcS-FM (*n* = 49)		*P* value
	Mean (SD)	*n* (%)	Mean (SD)	*n* (%)	
Age (years)	40.5 (5.9)		40.6 (5.3)		0.931^b^
BMI (kg/m^2^)	23.6 (2.7)		22.8 (2.8)		0.184^b^
No. living with a child^a^		17 (36.2)		19 (38.8)	0.792^c^
No. of smokers		9 (19.1)		16 (32.7)	0.306^c^
SIgA secretion rate (μg/min)	57.4 (23.7)		50.8 (31.0)		0.245^b^
Product compliance (%)	99.7 (1.1)		99.7 (0.9)		0.930^b^

*CM* control milk, *LcS-FM* *L. casei* strain Shirota-fermented milk, *BMI* body mass index, *SIgA* salivary immunoglobulin A

^a^No. of participants living with one or more children attending elementary/junior high school

^b^ *P* values analyzed by the unpaired Student’s *t* test

^c^ *P* values analyzed by the Chi-square test

### Incidence of URTIs

**Fig. 2:**
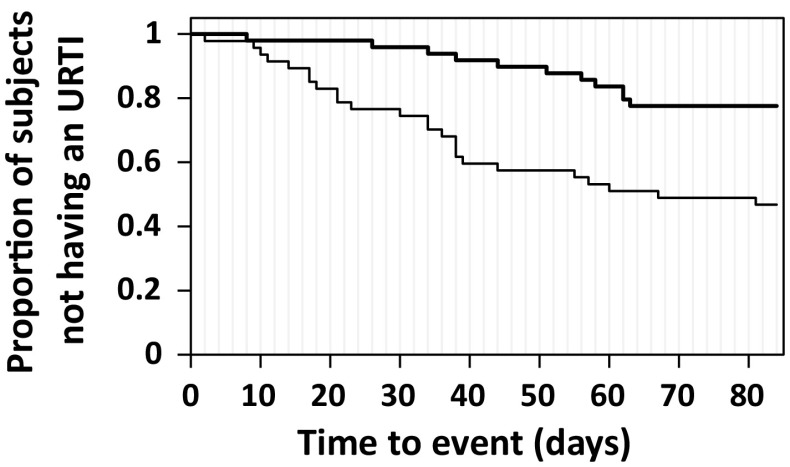
Kaplan–Meier time-to-event curves for the first URTI. The URTI-free rates were 0.78 (95 % CI 0.66–0.89) and 0.47 (95 % CI 0.33–0.61) in the LcS-FM (*thick line*) and the CM (*thin line*) groups, respectively

The primary outcome measure of this study was URTI incidence, the results of which are shown in Table 2. The incidence rates of URTIs during the intervention period (weeks 1–12) were significantly different at 53.2 and 22.4 % for the CM and LcS-FM groups, respectively (*P* = 0.002). The incidence rate of the common cold was significantly lower in the LcS-FM group than in the CM group (18.4 vs. 44.7 %, *P* = 0.005). The incidence of influenza was 10.6 % in the CM group and 4.1 % in the LcS-FM group, although this difference did not reach statistical significance. Time-to-episode curves for the first URTI are shown in Fig. 2. The curve of the LcS-FM group was significantly higher than that of the CM group over the test period (log-rank test: *χ* ^2^ 11.25, *P* = 0.0008). The URTI-free rates were 0.78 (95 % CI 0.66–0.89) and 0.47 (95 % CI 0.33–0.61) in the LcS-FM group and the CM group, respectively. The hazard ratio was calculated 0.32 (95 % CI 0.16–0.65).

**Table 2:** Primary outcomes: incidence of URTIs

	CM (*n* = 47)	LcS-FM (*n* = 49)	*P* value^b^
	*n* ^a^ (%)	*n* ^a^ (%)	
Whole period (1–12 weeks)			
URTIs	25 (53.2)	11 (22.4)	0.002
Common cold	21 (44.7)	9 (18.4)	0.005
Influenza	5 (10.6)	2 (4.1)	0.201
1st period (1–4 weeks)			
URTIs	11 (23.4)	3 (6.1)	0.017
Common cold	11 (23.4)	3 (6.1)	0.017
Influenza	0 (0.0)	0 (0.0)	–
2nd period (5–8 weeks)			
URTIs	12 (25.5)	5 (10.2)	0.049
Common cold	10 (21.3)	4 (8.2)	0.069
Influenza	3 (6.4)	1 (2.0)	0.293
3rd period (9–12 weeks)			
URTIs	7 (14.9)	6 (12.2)	0.705
Common cold	5 (10.6)	5 (10.2)	0.603
Influenza	2 (4.3)	1 (2.0)	0.484

*URTI* upper respiratory tract infection, *CM* control milk, *LcS-FM* *L. casei* strain Shirota-fermented milk

^a^No. of participants with URTIs/common cold/influenza

^b^ *P* values analyzed by the Chi-square test; *P* < 0.05 is considered statistically significant

We further analyzed the incidence rates of URTIs during the following three periods: 1st period (weeks 1–4); 2nd period (weeks 5–8); 3rd period (weeks 9–12). As shown in Table 2, the incidence of URTIs was significantly reduced in both the 1st and the 2nd periods (*P* = 0.017 and *P* = 0.049, respectively) but not in the 3rd period in the LcS-FM group.

### Characteristics of URTI episodes

The effects of the test drinks on the cumulative number of episodes, the cumulative days with symptoms, and the duration and severity of URTIs were analyzed. The cumulative numbers of URTI episodes per person during the intervention period were 0.7 in the CM group and 0.3 in the LcS-FM group (*P* = 0.004; Table 3). The LcS-FM group had fewer cumulative days with URTI symptoms per person during the test period, and the duration of each URTI episode was shorter compared with the CM group (*P* = 0.001 and *P* = 0.002, respectively). The mean and peak daily severity scores of URTIs did not differ between the two groups.

**Table 3:** Secondary outcomes: episode number per person, total days with symptoms per person, duration and severity of URTIs

	CM (*n* = 47)	LcS-FM (*n* = 49)	*P* value
	Mean (SD)	Mean (SD)	
Cumulative number of URTI episodes	0.7 (0.7)	0.3 (0.8)	0.004^a^
Cumulative days with symptoms (days)	3.4 (4.3)	1.0 (2.1)	0.001^b^
Duration per episode (days)	5.0 (2.5)	2.8 (1.6)	0.002^b^
Mean severity score^c^	15.8 (2.8)	15.9 (2.6)	0.966^a^
Peak severity score^d^	18.2 (4.2)	17.4 (3.6)	0.882^a^

*URTI* upper respiratory tract infection, *CM* control milk, *LcS-FM* *L. casei* strain Shirota-fermented milk

^a^ *P* values analyzed by the Wilcoxon two-sample test; *P* < 0.05 is considered statistically significant

^b^ *P* values analyzed by the unpaired Student’s *t* test; *P* < 0.05 is considered statistically significant

^c^Mean values of daily severity score during the period of each URTI episode

^d^Peak values of daily severity score of URTIs

### Blood and saliva parameters

**Fig. 3:**
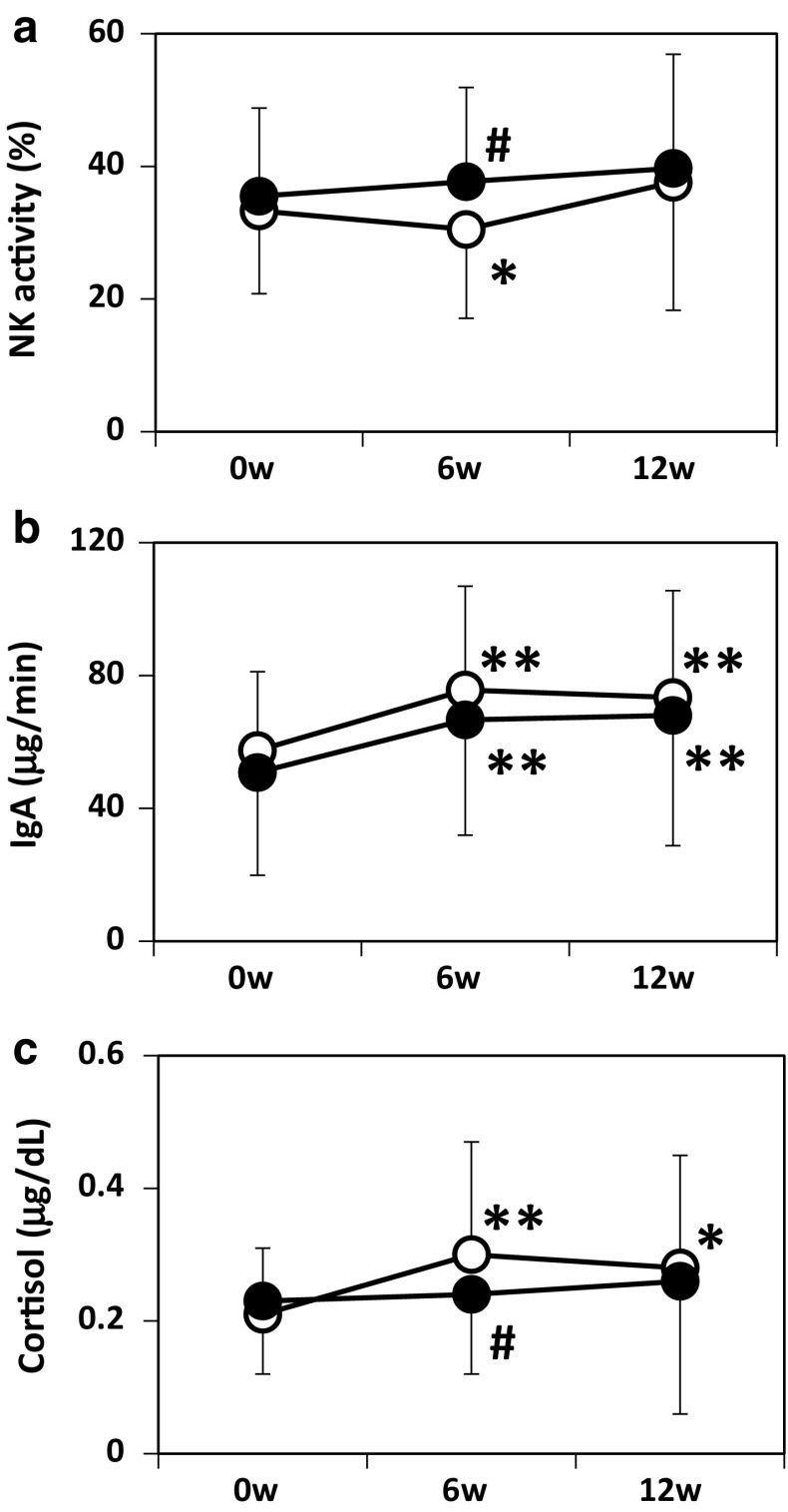
Changes in NK cell activity in peripheral blood mononuclear cells, IgA secretion rate, and cortisol levels in saliva throughout the intervention period of 12 weeks. Data are expressed as means ± SD. *Open circles*, CM group (*n* = 47); *closed circles*, LcS-FM group (*n* = 49). *P < 0.05; **P < 0.01 versus week 0 in the same group; ^#^ *P* < 0.05 versus CM group

Since immune defense mechanisms are important for host resistance to viral infections, we measured NK cell activity in peripheral blood mononuclear cells and IgA secretion rates in saliva at weeks 0, 6, and 12 of the intervention period. NK cell activity decreased at week 6 compared with week 0 in the CM group, but not in the LcS-FM group (Fig. 3). Thus, NK cell activity was significantly higher at week 6 in the LcS-FM group than in the CM group (*P* = 0.013). Salivary IgA secretion increased in weeks 6 and 12 in both groups, and the secretion did not differ between the groups.

Salivary levels of the stress marker cortisol were analyzed. In contrast to the change in NK cell activity, cortisol levels were increased at week 6 in the CM group but not in the LcS-FM group, and the difference was significant between the groups (*P* = 0.045).

### Safety

There were no adverse effects associated with consumption of the test drinks during the test period, as confirmed by the physician following examinations of blood and urine, physiological tests, analysis of health diaries, and interviews (data not shown).

## Discussion

This randomized controlled trial conducted in healthy middle-aged male office workers clearly demonstrated that the daily intake of probiotic fermented milk, LcS-FM, reduced the risk of URTIs. The time to first URTI episode analysis showed that the risk reduction efficacy for LcS-FM was 68 % (95 % CI 35–84 %) compared with CM. The analysis of URTI incidence for divided three periods showed that the positive health effects of LcS-FM were more evident in earlier test periods. The incidence of the common cold in the LcS-FM group (18.4 %) during the intervention period of 12 weeks decreased by more than half compared with the control group (44.7 %). The reduction in the incidence of influenza did not reach statistical significance, which might be due to the low incidence rates of both groups (10.6 % for the control; 4.1 % for the LcS-FM group). In addition to the reduced risk for URTIs, LcS-FM reduced the cumulative number of days with URTI symptoms and shortened the duration of each episode.

Intervention studies using similar LcS-FM drinks against URTIs have been conducted previously. The results obtained from two previous trials in elderly participants aged over 80 years did not show a significant reduction in URTI incidence rates [24, 25]. In contrast, the present study clearly demonstrated that LcS-FM effectively reduced the risk of URTIs. These results are consistent with the results of a trial conducted in athletes aged 18–55 years [20]. The ages of the participants in the latter study were similar to those in the present study, and thus, the different age groups among these studies might explain the conflicting results.

Another difference between the present and the previous studies should be noted. The viable number of LcS cells ingested daily was a minimum of 1.0 × 10^11^ cells in this study, while in the aforementioned studies with elderly participants, the numbers of viable cells were 1.3 × 10^10^ or 4.0 × 10^10^. In general, a higher number of ingested viable probiotic cells will tend to lead to a more beneficial outcome. The dose–response associated with improved efficacy against URTIs has been reported. De Vrese et al. [28] showed that while the intake of a multivitamin and mineral tablet containing 5 × 10^7^ viable lactobacillus and bifidobacterium cells shortened the duration of common cold infections, it had no effect on the incidence rate; however, another study showed that the similar tablet containing 5 × 10^8^ viable probiotic cells reduced the incidence rate [29]. The relationship between viable LcS cell numbers and efficacy should be examined and discussed in future studies.

Many probiotic intervention studies that have demonstrated efficacy against URTIs have been conducted in infants, children, students, and the elderly [9–12]. Physically active individuals, including rugby players and athletes of endurance-based sports, might also be targets for probiotics [20, 27, 30]. Since immune defense activities are relatively weak in such populations, they generally experience more infections. The present study demonstrated that healthy middle-aged office workers are also a promising target population for probiotics. A clinical trial in employees at a manufacturing company in Sweden showed that daily consumption of *L. reuteri* ATCC 55730 reduced the incidence of sick leave due to respiratory or gastrointestinal illnesses during the 80 days of intervention [13]. The efficacy of probiotics was more evident in a subgroup of shift workers [13, 14]. The present study demonstrated the efficacy of probiotics in typical desk workers. In general, such employees work under pressure and stress every day, which might be a risk factor for lowered immune defenses. Daily consumption of certain probiotics at higher doses may prevent disturbances in immune function resulting from stressful events in daily life. Thus, these results suggested the potential use of probiotics to improve health in the workplace.

In the present study, LcS-FM consumption led to improvements in immunological parameters and a stress marker. At week 6 of the intervention period, NK cell activity decreased and the salivary levels of cortisol increased in the control group, but these parameters kept within baseline levels in the LcS-FM group. NK cells play a very important role in the prevention of viral infections, including URTIs [15]. Inhibition of reduced NK cell activity might increase resistance to URTIs. The reason for the changes in immunological parameters and stress markers in the control group is not clear. However, the participants were assigned unusual daily tasks, including the recording of health information of many parameters during the intervention period, which might have affected their immune and endocrine systems, especially in the first half of the intervention period. At a later period, the participants might no longer feel such stress because getting used to the daily tasks and the parameters might return to the baseline levels. If the case is true, intake of LcS-FM may prevent decrease in immune defense activities caused by certain events, rather than augment their baseline levels. This may be the reason why improvements in the parameters by LcS-FM intake were detected only at week 6. In fact, a previous study in healthy adults with the normal levels of NK cell activity showed that LcS intake had no effect on their NK cell activity [31].

Previous studies have revealed that the daily consumption of LcS-FM helps to recover low NK cell activity [18]. In vitro studies with human peripheral blood mononuclear cells suggest that LcS stimulates monocytes/macrophages to produce IL-12 and augments NK cell activity [32, 33]. The direct regulation of immune function by LcS might be realized in the present clinical trial, which in turn might reduce the incidence of URTIs.

LcS-FM may mediate another protective mechanism whereby the ingestion of the probiotic drink reduces stress-related host responses. In the present study, LcS-FM inhibited increases in the levels of the stress hormone cortisol, which has the ability to decrease NK cell activity [34]. Some clinical trials with probiotics or prebiotics have already focused on psychological stress-related decreases of host defense mechanisms. The prevention of URTIs by probiotic or prebiotic supplementation among university students under stress due to final exams was reported [35, 36]. However, in contrast to the present study, the levels of cortisol were not examined in these trials.

Increasing evidence suggests that probiotics and the intestinal microbiota affect the nervous system and brain function [37]. Recently, Tanida et al. [38, 39] showed that some probiotic strains including LcS suppress the neural activity of sympathetic nerves in rats. The nervous system is closely related to the immune system [40]. Future studies should focus on nervous system-related actions of probiotics as well as their direct immune modulatory activities to obtain a better understanding of the precise mechanisms of preventing viral infections via probiotics.

In conclusion, the findings obtained in this study suggest that the daily intake of fermented milk with LcS may reduce the risk of URTIs in healthy middle-aged office workers, probably through modulation of the immune system.
